# Expression of Concern: Neurotoxicity Induced by Bupivacaine via T-Type Calcium Channels in SH-SY5Y Cells

**DOI:** 10.1371/journal.pone.0222623

**Published:** 2019-09-12

**Authors:** The PLOS ONE Editors

Following publication of this article [1] concerns were raised regarding images in Figs 5 and 7. Specifically,

- In Fig 5A there are regions of similarity between the dot plots in the top left panel “S” and the top middle panel “S+NNC100”.
- In Fig 5A there are regions of similarity between the dot plots in the bottom middle panel “S+B+NNC50” and the bottom right panel “S+B+NNC100”.
- In the western blot in Fig 7, there are horizontal discontinuities in the background shading above and below the band in lane 5 of the cleaved caspase-3 panel.
- In the western blot in Fig 7, there are similarities between bands in lanes 1–3 compared to lanes 4–6 of the β-actin panel.

The corresponding author has clarified that the original flow cytometry experiments were carried out by a technician in another laboratory within the same institution. The corresponding author stands by the flow cytometry dot plots in Fig 5 as generated from independent samples. The original raw data files for the flow cytometry experiments in Figs 4 and 5 are no longer available. Higher resolution image files of the original dot plots are provided here as Supporting Information (S1 File). The authors have repeated the experiment and provide a revised Fig 5 using the new data.

The underlying raw data files for the repeat experiment are provided as Supporting Information (S2 File).

The corresponding author stands by the western blot images in Fig 7 as accurate; however, the original uncropped blot images are not available.

The underlying data for the other parts of the article remain available upon request.

The *PLOS ONE* Editors issue an Expression of Concern to inform readers about the concerns regarding the accuracy of the western blot and flow cytometry image data in the original article, which could not be fully resolved due to the unavailability of the original raw data files.

**Fig 5:**
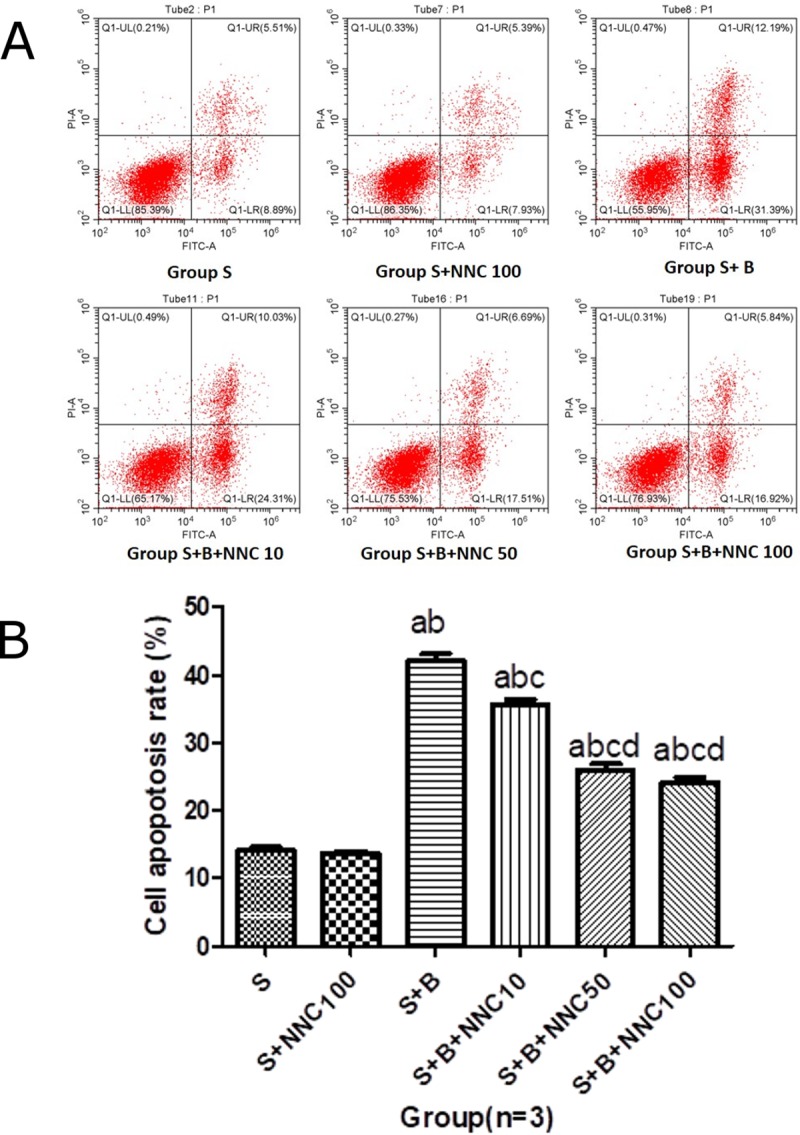
NNC 55–0396 dihydrochloride protects SH-SY5Y cells from bupivacaine-induced apoptosis. Cells were either treated with the indicated concentrations of NNC 55–0396 dihydrochloride or left untreated prior to 1 mM bupivaine treatment for 24 h. Apoptosis rate was measured with flow cytometry (%, mean±SD, n = 3). A: Representative image from the flow cytometric analysis. B: Rates of apoptosis in the different treatment groups. a P<0.05 vs. S group; b P<0.05 vs. S+NNC 100 group; c P<0.05 vs. S+B group; d P<0.05 vs. S+B+NNC 10 group.

## Supporting information

S1 File Higher resolution image files of the dot plots in the original Fig 5. (RAR)

S2 File The underlying data files for the repeat experiment in the revised Fig 5. (ZIP)
