# Supplementary figures and images for: Expression of Concern: Neurotoxicity Induced by Bupivacaine via T-Type Calcium Channels in SH-SY5Y Cells
Source: PLoS One. 2019 Sep 12;14(9):e0222623. doi: 10.1371/journal.pone.0222623 (PMC6742379; doi:10.1371/journal.pone.0222623)

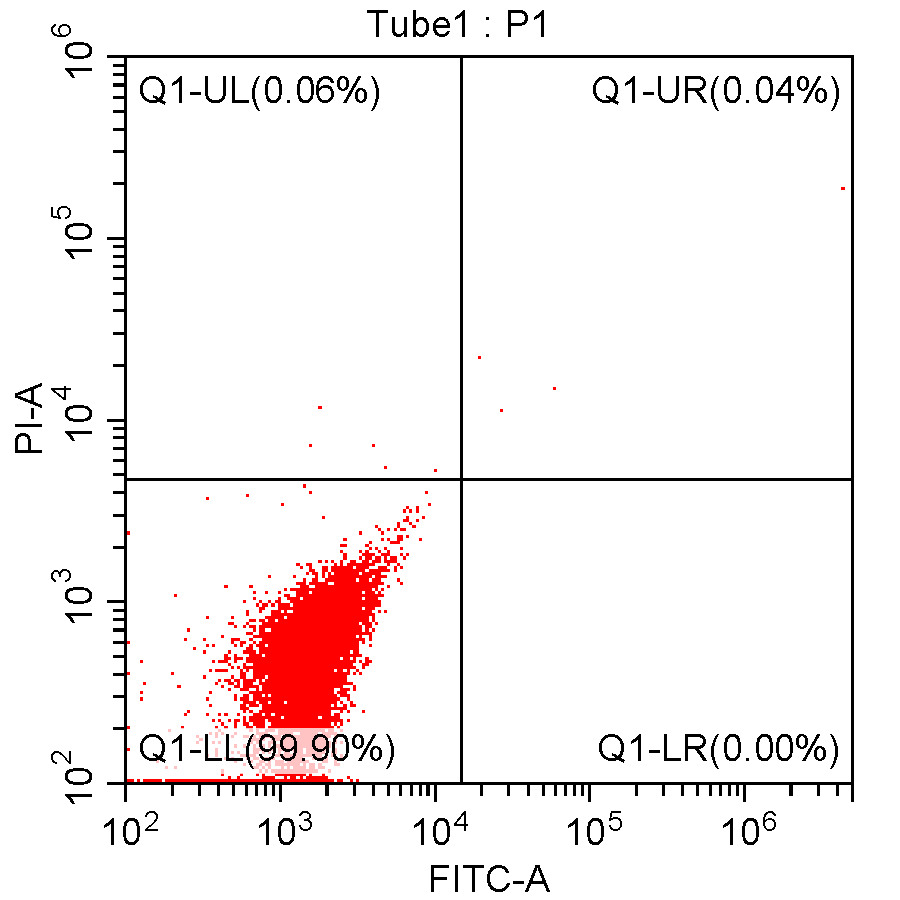

Supplement: S2 File — (ZIP) [file pone.0222623.s002.zip › S2 File/Gate Tube1_Plot1.bmp]
